# Altered Motoneuron Properties Contribute to Motor Deficits in a Rabbit Hypoxia-Ischemia Model of Cerebral Palsy

**DOI:** 10.3389/fncel.2020.00069

**Published:** 2020-03-25

**Authors:** Preston R. Steele, Clarissa Fantin Cavarsan, Lisa Dowaliby, Megan Westefeld, N. Katenka, Alexander Drobyshevsky, Monica A. Gorassini, Katharina A. Quinlan

**Affiliations:** ^1^Interdepartmental Neuroscience Program, University of Rhode Island, Kingston, RI, United States; ^2^George and Anne Ryan Institute for Neuroscience, University of Rhode Island, Kingston, RI, United States; ^3^Department of Biomedical and Pharmaceutical Sciences, College of Pharmacy, University of Rhode Island, Kingston, RI, United States; ^4^Department of Computer Science and Statistics, University of Rhode Island, Kingston, RI, United States; ^5^Northshore University Health System Research Institute, Evanston, IL, United States; ^6^Department of Biomedical Engineering, University of Alberta, Edmonton, AB, Canada; ^7^Department of Physiology, Northwestern University Feinberg School of Medicine, Chicago, IL, United States

**Keywords:** cerebral palsy, hypoxia-ischemia, frequency-current, persistent inward current, rabbit

## Abstract

Cerebral palsy (CP) is caused by a variety of factors attributed to early brain damage, resulting in permanently impaired motor control, marked by weakness and muscle stiffness. To find out if altered physiology of spinal motoneurons (MNs) could contribute to movement deficits, we performed whole-cell patch-clamp in neonatal rabbit spinal cord slices after developmental injury at 79% gestation. After preterm hypoxia-ischemia (HI), rabbits are born with motor deficits consistent with a spastic phenotype including hypertonia and hyperreflexia. There is a range in severity, thus kits are classified as severely affected, mildly affected, or unaffected based on modified Ashworth scores and other behavioral tests. At postnatal day (P)0–5, we recorded electrophysiological parameters of 40 MNs in transverse spinal cord slices using whole-cell patch-clamp. We found significant differences between groups (severe, mild, unaffected and sham control MNs). Severe HI MNs showed more sustained firing patterns, depolarized resting membrane potential, and fired action potentials at a higher frequency. These properties could contribute to muscle stiffness, a hallmark of spastic CP. Interestingly altered persistent inward currents (PICs) and morphology in severe HI MNs would dampen excitability (depolarized PIC onset and increased dendritic length). In summary, changes we observed in spinal MN physiology likely contribute to the severity of the phenotype, and therapeutic strategies for CP could target the excitability of spinal MNs.

## Introduction

Cerebral palsy (CP) is not well understood, despite its prevalence and seriousness. There exist only a few evidence-based treatments for CP: the effectiveness of many currently used therapeutic strategies is unclear (Novak et al., 2013; Wimalasundera and Stevenson, 2016). Recent clinical advances include use of magnesium sulfate and hypothermia after hypoxic-ischemic encephalopathy to acutely reduce neural damage (Yager et al., 1993; Thoresen et al., 1996; Magee et al., 2011; Rouse and Gibbins, 2013), but little basic research is devoted to addressing symptoms after they arise. Part of the problem in treating CP may be the diversity of causes including neonatal stroke, placental insufficiency, preterm birth, inflammation, traumatic injury, difficulties during birth and many other contributing factors (MacLennan and International Cerebral Palsy Task Force, 1999; Graham et al., 2016). Another problem could be that modeling the condition in animals is complicated, and while rodent models are useful for the development of neuroprotective strategies, larger animal models are needed to study motor deficits (Clowry et al., 2014; Cavarsan et al., 2019).

Loss of corticospinal control of movement is considered causative of motor deficits in CP, but little investigation into the precise effect on spinal circuits has been conducted. A notable exception is the work of John H. Martin and colleagues, who have documented changes in corticospinal synaptic connectivity in specific spinal laminae and loss of cholinergic interneurons after either cortical silencing or lesioning the corticospinal tract (Martin et al., 1999; Li and Martin, 2000; Friel and Martin, 2005, 2007; Friel et al., 2012; Jiang et al., 2016, 2018). Another important study showed changes in parvalbumin-positive spinal interneurons after cortical silencing in development (Clowry et al., 2004; Clowry, 2007). Both of these interneuron classes (parvalbumin-positive and cholinergic) are synaptically connected to spinal MNs and could contribute to altered motor output after developmental injury. Based on these foundational studies, we hypothesized that altering development with hypoxia-ischemia (HI) injury would also alter the development of MNs, specifically the electrophysiological properties governing excitability in spinal MNs. We further hypothesized that changes in excitability would correspond/contribute to the severity of motor deficits. In short, that altered activity of spinal MNs could contribute to muscle stiffness and spasticity.

To assess changes in the intrinsic properties of spinal MNs, we used the rabbit HI model of CP (Derrick et al., 2004). It’s been shown in previous studies that HI injury during late gestation in rabbits can result in a variety of neurologic and muscular damage, including muscle stiffness (Derrick et al., 2004), loss of neurons in cortical layers 3 and 5, white matter injury, thinning of the corticospinal tract (Buser et al., 2010), cell death in the spinal cord and decreased numbers of spinal MNs (Drobyshevsky and Quinlan, 2017), increased sarcomere length, decreased muscle mass and hyperreflexia (Synowiec et al., 2019). There is also an increase in spinal monoamines which could increase the excitability of spinal neurons and thus promote spasticity (Bellot et al., 2014; Drobyshevsky et al., 2015). Thus, changes observed in spinal MNs in the rabbit model could be directly compared to motor deficits.

Changes in MN physiology are likely to contribute to motor impairment in CP, yet this has not been directly assessed in any animal models. Thus, we assessed electrophysiological parameters in spinal MNs in neonatal rabbits after sham surgery or hypoxic-ischemic insult during development.

## Materials and Methods

All rabbits were used according to National Institutes of Health guide for the care and use of laboratory animals, and the University of Rhode Island’s, Northwestern University’s and Northshore University Health System’s Animal Care and Use Committee guidelines. Pregnant New Zealand White rabbits (Charles River Laboratories, Inc. Wilmington, MA, USA), underwent HI procedures as described previously (Derrick et al., 2004; Buser et al., 2010). Briefly, the procedure was performed at ~80% gestation (day 25 of gestation, or E25), a time when HI has been found to result in the greatest degree of white matter injury and corticospinal tract thinning. Dams were anesthetized, and the left femoral artery was isolated. A Fogarty balloon catheter was inserted into the femoral and advanced to the level of the descending aorta, above the uterine arteries and inflated for 40 min. Sham animals underwent the same procedures but without the inflation of the catheter. After the procedure, the dam recovered and later gave birth to kits with HI injuries. Categorization of the severity of the phenotype was performed by a blinded observer, using a modified Ashworth scale, observation/tests for activity, locomotion, posture, righting reflex, muscle tone (as described in Derrick et al., 2004). Kits could be given a maximum score of 6 (normal posture, righting and joint resistance). Since, there was a large variation in the severity of motor deficits, HI kits were divided into three groups: HI unaffected (scores were the same range as control kits, 5–6), HI mild (scores 3–4), HI severe (scores 1–2). One rabbit kit which was affected by HI but displayed a phenotype of hypotonia instead of hypertonia was removed from the data set. All other kits included in this study displayed a hypertonic phenotype if affected by HI.

### Patch Clamp

Whole-cell patch-clamp was performed similarly to previously published work (Quinlan et al., 2011) from P0–5. Briefly, horizontal spinal cord slices 350 μm thick were obtained using a Leica 1,000 vibratome. Slices were incubated for 1 h at 30°C and recordings were performed at room temperature. During recording, slices were perfused with oxygenated (95% O_2_ and 5% CO_2_) modified Ringer’s solution containing (in mM): 111 NaCl, 3.09 KCl, 25.0 NaHCO_3_, 1.10 KH_2_PO_4_, 1.26 MgSO_4_, 2.52 CaCl_2_, and 11.1 glucose at 2 ml/min. Whole-cell patch electrodes (1–3 MΩ) contained (in mM) 138 K-gluconate, 10 HEPES, 5 ATP-Mg, 0.3 GTP-Li and Texas Red dextran (150 μM, 3,000 MW). PICs were measured in voltage-clamp mode with holding-potential of −90 mV and depolarizing voltage ramps of both 36 mV/s and 11.25 mV/s bringing the cell to 0 mV in 2.5 s or 8 s, respectively and then back to the holding potential in the following 2.5 s or 8 s. Input resistance was measured from the slope of the leak current near the holding potential. Capacitance was measured with Multiclamp’s whole-cell capacitance compensation function. Resting membrane potential was measured in voltage-clamp as the voltage at which there is 0 pA of injected current in the descending ramp. In current clamp, frequency—current measurements were obtained from current ramps. The first spike on the current ramp was used to measure the properties of action potentials. The threshold voltage was defined as the voltage at which the action potential slope exceeds 10 V/s. Rate of rise and fall of the action potential were measured as peak and trough of the first derivative of the action potential. The duration of the action potential was measured at half-peak (defined as the midpoint between overshoot and threshold voltages). Depolarizing current steps of varying amplitude were used to find maximum firing rates (near depolarization block) and to measure after-spike afterhyperpolarization (in single spikes elicited near-threshold). Hyperpolarizing current steps (typically between −850 and −1,250 pA) were used to measure hyperpolarization-activated sag currents (I_H_). Neuron selection: neurons were targeted in MN pools mainly from cervical and lumbar regions of the cord and were removed from the data set if their resting membrane potential was more depolarized than −45 mV in current clamp.

### Imaging

**Figure 1:**
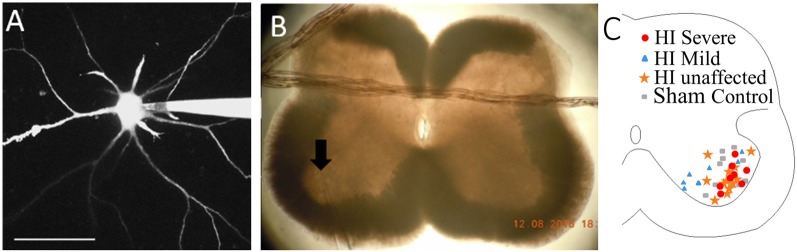
Patch clamp of spinal motoneurons (MNs) with dye-filling *via* patch electrode as shown in panel **(A)**. Scale bar = 100 μm. **(B)** Placement of patch electrode (at arrow) within the slice is captured with a photo. **(C)** Map of recorded MNs within medial and lateral motor pools. Red circles = HI Severe, blue triangles = HI Mild, yellow stars = HI unaffected, gray rectangles = Sham Controls.

After electrophysiological measurements were obtained, MNs were imaged to assess anatomical development, and photos were obtained of the electrode placement within the spinal cord slice, as shown in Figure 1. Images were acquired with a Nikon microscope fitted with a 40× water-dipping objective lens and two-photon excitation fluorescence microscopy performed with a galvanometer-based Coherent Chameleon Ultra II laser. To optimize the excitation of red/green fluorophores, the laser was tuned to 900 nm. 3D reconstructions of MNs were created using Neurolucida 360° software. It is likely that some processes extended past the surface of the slice and were excluded from reconstructions. However, since this was the case for all MNs in this study it is unlikely to have an impact on the findings.

### Statistics

All variables were checked for normality and homogeneity (using Shapiro–Wilk and Levene’s tests). The variables that were parametric (normal and homogenous) were run with one-way Analysis of Variance (ANOVA) and then assessed *post hoc* with a Tukey test for between-group significance. The non-parametric variables were run with the Kruskal–Wallis test followed by Dunn’s test as a *post hoc* analysis to assess significance between groups, adjusting the *p*-value for multiple comparisons. The analysis was done using R software for determining the significance of parameters over groups, according to their injury classification (sham, HI unaffected, HI mildly affected, and HI severely affected). Injury classification, age of the kit (P0–P5), and spinal cord region (cervical, thoracic, lumbar or sacral) were all tested. Significance was determined by *p*-values ≤ 0.05.

## Results

After HI surgery was performed in pregnant dams at 79% gestation, kits were born naturally about a week later. At ages P0, neonates were rated as unaffected, mildly affected or severely affected. Since, there was a large variation in the severity of motor deficits, HI kits were divided into three groups: HI unaffected, HI mild, HI severe. Experiments were all performed in the first 5 days of life. Over 40 spinal MNs were patched in transverse spinal cord slices, and over 40 parameters were measured from each. To determine the significance of the variables, a one-way ANOVA was performed to find differences among three factors: (1) injury classification (sham control, HI unaffected, HI mildly affected, and HI severely affected); (2) age (postnatal day 0–5); and (3) spinal region (cervical through sacral). All data, including mean, standard deviation, group size, and the *p*-value is included in table format (Tables s 1–5 and Supplementary Tables S1, S2).

**Table 1:** Frequency—current characteristics.

Variable	Condition	Mean	*SD*	*N*	*p*
I_ON_ (pA)	Sham	213	149	9
	HI Unaffected	627	475	12	0.129
	HI Mild	422	458	10	0.507
	HI Severe	459	375	8	0.450
I_OFF_ (pA)	Sham	416	357	9
	HI Unaffected	768	538	12	0.545
	HI Mild	509	319	10	1.000
	HI Severe	443	452	8	1.000
Δ I (pA)	Sham	219	224	9
	HI Unaffected	141	159	12	0.496
	HI Mild	88	279	10	0.106
	HI Severe	−16	84	8	0.046*
FI Slope (Hz/nA)	Sham	23	18	9
	HI Unaffected	13	4	12	0.557
	HI Mild	23	23	10	0.945
	HI Severe	20	9	8	0.370
Threshold (mV)	Sham	−35.8	7.2	9
	HI Unaffected	−32.2	8.0	12	0.279
	HI Mild	−36.3	7.1	10	0.879
	HI Severe	−29.9	6.7	8	0.107
Maximum instantaneous firing frequency (Hz)	Sham	98	28	9
	HI Unaffected	106	32	12	0.556
	HI Mild	129	33	10	0.025*
	HI Severe	129	23	8	0.045*
Maximum steady state firing frequency (Hz)	Sham	30	10	9
	HI Unaffected	36	13	12	0.552
	HI Mild	45	18	10	0.346
	HI Severe	37	13	8	0.559
Resting membrane potential (mV)	Sham	−60.8	6.4	9
	HI Unaffected	−53.9	11.3	13	0.070
	HI Mild	−58.5	4.3	10	0.556
	HI Severe	−50.5	9.1	8	0.017*

*Significant difference to sham animals.

**Table 2:** Persistent inward current characteristics (5 s ramp).

Variable	Condition	Mean	*SD*	*N*	*p*
Norm PIC amp (pA/pF)	Sham	−2.10	2.44	9
	HI Unaffected	−1.45	0.81	13	0.852
	HI Mild	−1.86	1.02	9	0.988
	HI Severe	−1.22	0.41	8	0.923
PIC onset (mV)	Sham	−42.9	7.3	9
	HI Unaffected	−37.4	6.7	13	0.200
	HI Mild	−38.7	8.1	9	0.337
	HI Severe	−38.1	10.6	8	0.278
PIC max (mV)	Sham	−30.0	7.3	9
	HI Unaffected	−22.9	7.6	13	0.081
	HI Mild	−22.6	7.4	9	0.066
	HI Severe	−23.1	9.2	8	0.093
PIC negative slope range (mV)	Sham	12.9	3.1	9
	HI Unaffected	14.4	2.7	13	0.458
	HI Mild	16.0	2.8	9	0.250
	HI Severe	15.0	3.1	8	0.462
PIC amplitude (pA)	Sham	−280	185	9
	HI Unaffected	−366	195	13	0.344
	HI Mild	−417	273	9	0.162
	HI Severe	−344	115	8	0.525
Capacitance (pF)	Sham	192	98	9
	HI Unaffected	259	45	13	0.167
	HI Mild	214	77	10	0.471
	HI Severe	282	0	8	0.028*
Input resistance (MΩ)	Sham	60	35	9
	HI Unaffected	40	16	13	1.00
	HI Mild	56	34	10	1.00
	HI Severe	54	30	8	0.965

*Significant difference to sham animals.

**Table 3:** Persistent inward current characteristics (16 s ramp).

Variable	Condition	Mean	*SD*	*N*	*p*
Norm PIC amp (pA/pF)	Sham	−2.32	2.77	6
	HI Unaffected	−1.58	0.90	13	0.953
	HI Mild	−1.73	0.94	9	0.706
	HI Severe	−1.03	0.50	8	0.735
PIC onset (mV)	Sham	−47.2	6.0	6
	HI Unaffected	−39.7	6.3	13	0.086
	HI Mild	−40.9	9.7	9	0.169
	HI Severe	−35.0	11.5	8	0.013*
PIC max (mV)	Sham	−27.8	9.4	6	
	HI Unaffected	−21.4	7.4	13	0.175
	HI Mild	−24.2	9.9	9	0.479
	HI Severe	−18.3	11.1	8	0.071
PIC negative slope range (mV)	Sham	19.5	6.0	6
	HI Unaffected	18.3	3.2	13	0.927
	HI Mild	16.6	2.4	9	1.000
	HI Severe	16.7	4.1	8	0.872
PIC amplitude (pA)	Sham	−315	189	6
	HI Unaffected	−389	227	13	0.215
	HI Mild	−397	254	9	0.310
	HI Severe	−323	113	8	0.802

*Significant difference to sham animals.

**Table 4:** Soma morphology characteristics.

Variable	Condition	Mean	*SD*	*N*	*p*
Largest cross-sectional area (μm^2^)	Sham	577	316	9
	HI Unaffected	756	287	12	1.000
	HI Mild	676	307	10	0.277
	HI Severe	694	338	8	1.000
Surface area (μm^2^)	Sham	2,599	1,153	9
	HI Unaffected	3,609	2,083	12	0.591
	HI Mild	2,489	1,129	10	0.447
	HI Severe	2,582	851	8	0.155
Volume (μm^3^)	Sham	12,306	8,219	9
	HI Unaffected	23,735	28,313	12	0.716
	HI Mild	12,445	7,963	10	0.255
	HI Severe	12,264	6,308	8	0.826

**Table 5:** Dendrite characteristics.

Variable	Condition	Mean	*SD*	*N*	*p*
Number of dendrites	Sham	7.7	2.8	9
	HI Unaffected	9.5	1.4	12	0.089
	HI Mild	10.1	2.9	10	0.360
	HI Severe	10.1	2.5	8	0.128
Nodes	Sham	14.8	14.1	9	
	HI Unaffected	14.7	11.5	12	0.909
	HI Mild	13.5	5.7	10	0.800
	HI Severe	17.0	8.9	8	1.000
Length (μm)	Sham	1,103	649	9
	HI Unaffected	1,422	664	12	0.346
	HI Mild	1,443	537	10	0.591
	HI Severe	1,744	521	8	0.050*
Mean length (μm)	Sham	140	67	9
	HI Unaffected	149	67	12	0.947
	HI Mild	144	32	10	0.992
	HI Severe	178	57	8	0.169
Surface area (μm^2^)	Sham	6,264	3,842	9
	HI Unaffected	9,667	7,000	12	0.649
	HI Mild	7,537	3,293	10	0.526
	HI Severe	9,868	5,384	8	0.698
Mean surface area (μm^2^)	Sham	792	414	9
	HI Unaffected	1,024	772	12	0.919
	HI Mild	740	159	10	0.537
	HI Severe	1,012	603	8	0.612
Volume (μm^3^)	Sham	3,614	2,642	9
	HI Unaffected	6,926	7,512	12	0.807
	HI Mild	4,073	2,406	10	1.000
	HI Severe	5,844	5,239	8	0.664
Mean volume (μm^3^)	Sham	451	295	9
	HI Unaffected	743	836	12	0.771
	HI Mild	390	129	10	0.847
	HI Severe	605	587	8	0.917

*Significant difference to sham animals.

### HI MNs Show Sustained Firing and Higher Firing Frequency

**Figure 2:**
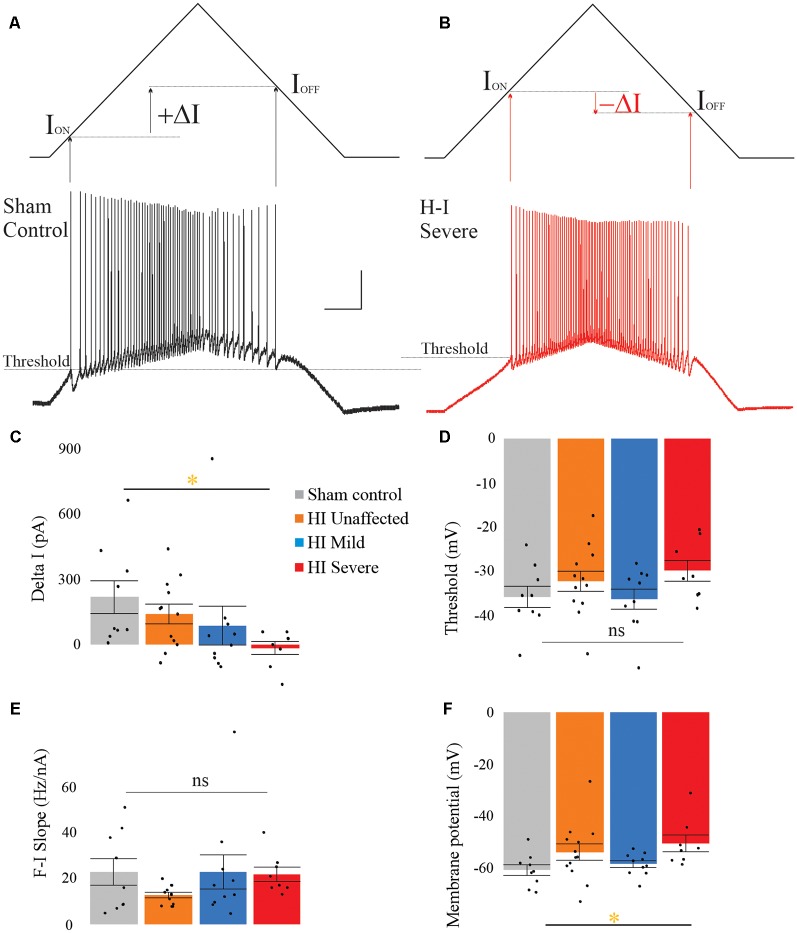
Severe HI MNs show more sustained firing than sham control MNs, and a more depolarized resting membrane potential. Control MNs (**A**) have larger values for ΔI compared to severe HI **(B)**. Average ΔI and threshold are shown in panels **(C)** and **(D)** for all groups. The frequency current relationship **(E)** was not significantly different between groups. Resting membrane potential was significantly more depolarized in HI severe MNs than sham control MNs **(F)**. Error bars = SEM. Scale bars in **(A)** = 20 mV (vertical) and 0.5 s (horizontal) and applies to panels **(A,B)**.

**Figure 3:**
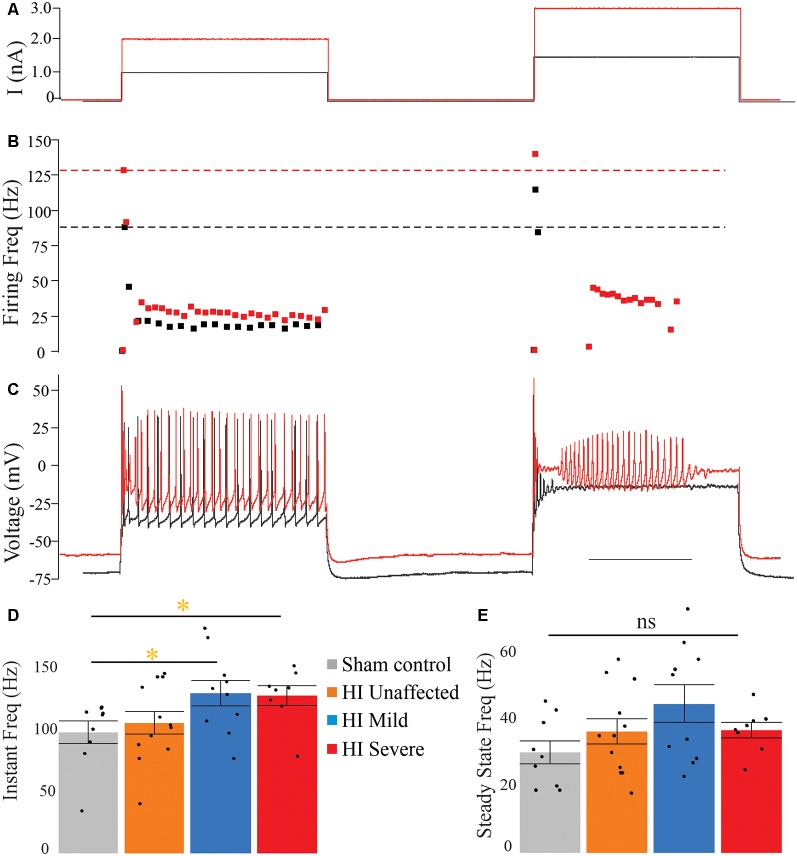
Instantaneous firing frequency is increased in severe HI MNs. Depolarizing current steps **(A)** evoked action potentials **(C)** in a typical sham control (black trace) and severe HI (red trace) MNs. **(B)** Dotted lines show peak firing frequency of MNs in panel **(C)**. **(D)** Average instantaneous and **(E)** steady-state firing frequency are shown for all groups. Error bars = SEM. Scale bar in **(C)** = 0.5 s and applies to panels **(A–C)**.

In rabbit kits severely injured by HI, MNs had significantly increased sustained firing. The frequency current (F-I) relationship was measured using the current ramps, as shown in Figure 2. Depolarizing current ramps are used to evoke firing, and current at onset and offset of firing (I_ON_ and I_OFF_) determine ΔI. In sham control MNs, ΔI was larger and always a positive value, indicating firing ceased at a higher current amplitude on the descending ramp than the current level that elicited firing on the ascending ramp (see Figure 2A). Severe HI MNs had a smaller, and usually negative ΔI, revealing increasingly sustained firing (see Figure 2B). Also, resting membrane potential was significantly more depolarized in HI MNs than sham controls (see Figure 2F). Another significant difference in severe HI MNs is the instantaneous firing rate, as shown in Figure 3. At the start of a depolarizing current step, the peak (instantaneous) firing rate is higher in severe HI MNs than sham controls (see Figure 3B). Sustained firing is also apparent in Figure 3C, in the second current step which evokes a brief burst of action potentials followed by depolarization block in both MNs. The severe HI MN recovers and resumes firing while the sham control MN remains in depolarization block. Mean values for both instantaneous and steady-state parameters are shown in bar graphs (Figures 3D,E). Significant changes in MN physiology were present in severely affected animals: *post hoc* analysis showed significant changes between sham and severe HI MNs in ΔI, RMP, and instantaneous firing rate. The instantaneous firing rate also reached significance in mild HI MNs vs. sham. No significant changes were present in HI unaffected MNs in any properties. While these parameters (ΔI, RMP, and instantaneous firing rate) suggest increased excitability in HI-injured MNs, there was no significant change in the threshold, I_ON_, or I_OFF_ in HI MNs. Thus, MNs from severely affected kits should not be classified as hyperexcitable *per se*, since they begin firing with the same depolarizing input and at the same voltage threshold.

### Changes in Spike Properties and Subthreshold Responses Were Not Present

A complete analysis of I_H_ (sag and rebound currents), action potential parameters, and after-spike after-hyperpolarization (AHP) was performed and no significant differences in these parameters were found between groups. All data is included in Supplementary Tables S1, S2.

### Persistent Inward Currents Suggest Excitability Is Dampened After HI

**Figure 4:**
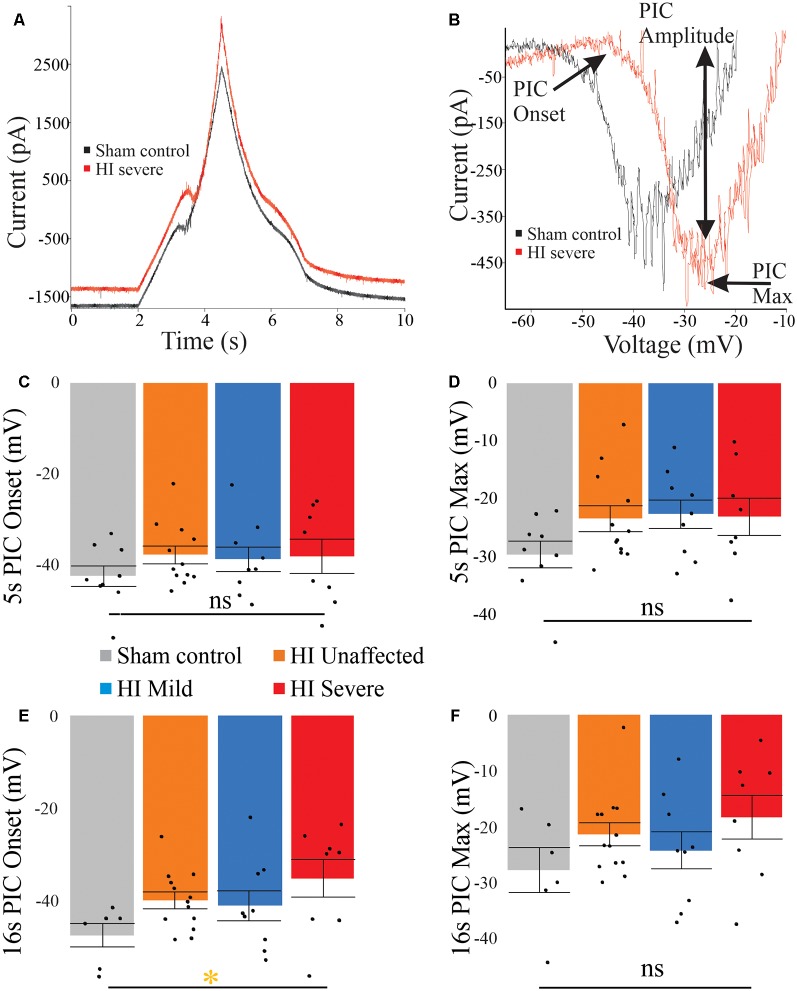
PICs are altered in HI injured MNs. **(A)** The typical current response to a 5-s voltage ramp in a sham (black trace) and HI severe (red) MN. **(B)** Leak-subtracted PICs from panel **(A)** are similar in amplitude. There is a trend for depolarized PIC onset **(C)** in short ramps after HI injury, which reaches significance in long ramps **(E)**. PIC Max did not reach significance **(D)** and **(F)**. Error bars = SEM.

PICs were significantly affected by hypoxia-ischemia (HI), revealing that intrinsic excitability may be dampened. PICs were measured using both short (5 s) and long (16 s) protocols, which can preferentially activate and inactivate Na^+^ and Ca^2+^ mediated PICs. The different protocols yielded different results. For example, as shown in Figure 4, voltage dependence (PIC onset and PIC Max) was unchanged in the PICs evoked using a short 5-s voltage ramp HI severe MNs. Using longer voltage ramps (16 s), PIC onset was significantly depolarized in HI severe MNs compared to sham (see Tables 2, 3). Since the change in PIC onset was more pronounced in longer ramps this could suggest an altered balance of Na^+^ and Ca^2+^ channel activation or altered activation/inactivation of these channels (see “Discussion” section). Change in the magnitude of the PIC was not observed outright in either of the protocols: the magnitude of the currents was similar between groups (see Figure 4 and Tables 2, 3). Intrinsic properties including capacitance (which significantly increased in HI MNs compared to sham) and input resistance of all MNs are included in Table 2.

### Morphology Affected by HI Injury

**Figure 5:**
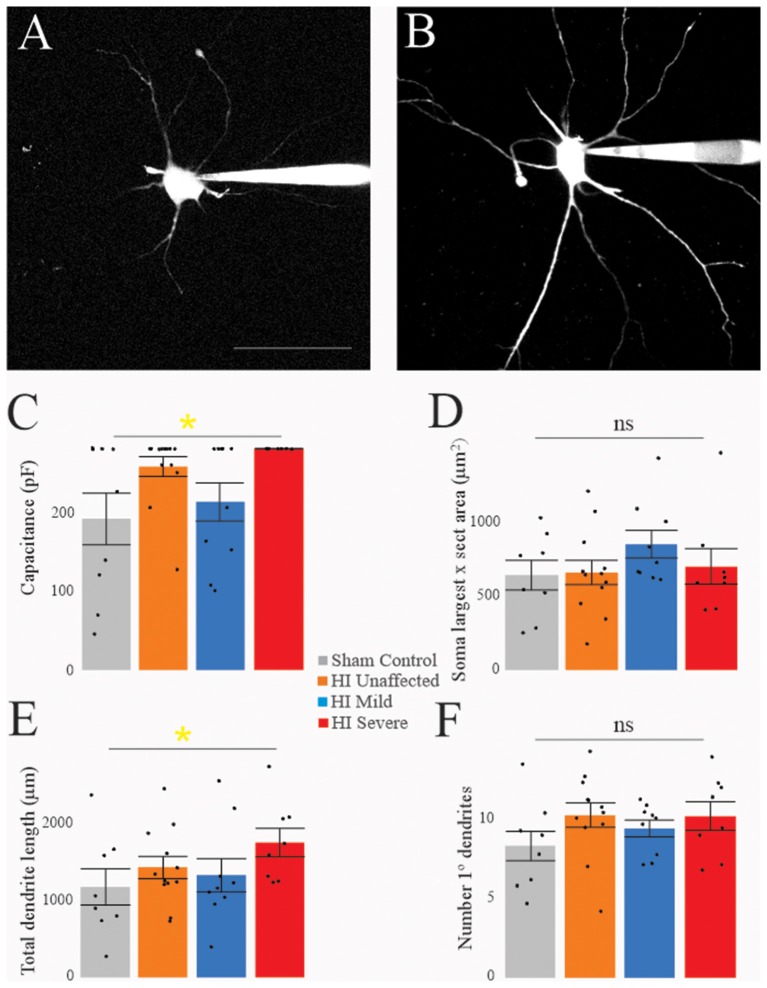
Morphology is affected by HI. Typical sham control **(A)** and HI severe **(B)** MNs filled with dye during patch-clamp (electrodes visible on right). Average values of whole-cell capacitance **(C)**, soma largest cross-sectional area **(D)**, total dendrite length **(E)**, and number of stem dendrites **(F)** are included for all neurons. Scale bar in **(A)** = 100 μm applies to **(A,B)**. Error bars = SEM.

Morphology of MNs was assessed in all patched neurons, as shown in Figure 5. As suggested by the significantly larger whole-cell capacitance, there were changes in MN morphology after HI injury. The soma size was unchanged: there were no significant differences between groups in soma’s largest cross-sectional area (Figure 5D) or other measurements of soma size (Table 4). There was, however, a significant increase in dendrite length in HI injured MNs compared to sham controls (Figure 5E), which could account for changes in electrical properties. Since, we recorded from motor pools throughout the spinal cord (cervical through sacral), there was a large amount of variability within our data set. Future studies in our lab focus on the analysis of specific motor pools. All data on dendritic morphology is included in Table 5.

### Age and Spinal Region

No significant changes in MN properties were found due to the spinal region. Postnatal age had a significant effect on the following properties: input resistance (decrease with age), action potential size (mV), rate of rise and rate of fall (all increase with age), 5 s PIC amplitude (increase with age), and normalized PIC (PIC/Cap; current density increased with age for both 5 and 16 s ramps). These results are in line with previous studies on embryonic and postnatal maturation of MNs.

## Discussion

### Summary

Electrophysiological properties of spinal MNs are altered by developmental HI injury, and the magnitude of changes is correlated to the severity of motor deficits. Specifically, these changes include increased sustained firing and a higher firing rate. These changes could indicate increased excitability and would contribute to muscle stiffness that is common in spastic CP. However, concomitant changes in PIC onset and longer dendritic length could serve to dampen excitability and could contribute to weakness. Since traditional views of CP largely view motor dysfunction as a result of the damaged motor cortex improperly signaling to spinal neurons, our new evidence suggests this is only part of the problem. Spinal MNs are not developing the same after HI and show an overall change in intrinsic properties. Whether these changes are directly due to the HI insult or indirectly due to downstream effects must be determined by future work.

### Contribution of Spinal Motoneurons to Dysfunction in Cerebral Palsy

Here, we show that MNs show altered excitability after HI injury, including elevated resting potential, and more sustained firing. Previous work showed that after HI injury in rabbits there were also fewer spinal MNs, and spinal interneurons in lamina VII were undergoing apoptosis (Drobyshevsky and Quinlan, 2017). After the loss of corticospinal projections, it was recently found that the spinal cholinergic interneurons which give rise to C boutons on MNs are lost (Jiang et al., 2016, 2018, 2019). Taken together this data suggests spinal circuits are: (1) just as vulnerable to HI injury as the developing cortex; and (2) potentially functioning with fewer neurons and altered circuitry. In addition to fewer neurons, there is also atrophy of the muscles which could contribute to motor deficits in CP. In mice, rabbits, and humans, muscle atrophy appears along with losses in numbers of MNs (Han et al., 2013; Marciniak et al., 2015; Drobyshevsky and Quinlan, 2017; Brandenburg et al., 2018). Recent work has shown similar changes to muscle architecture in the rabbit HI model of CP to humans, including atrophy, muscle shortening, and longer sarcomere length. Increased muscle stiffness in rabbits affected by HI was found even after administration of anesthetic—indicating some muscle stiffness is derived from mechanical changes in the muscles, though a large component of the muscle stiffness was diminished with anesthetic thus was driven neurally (Synowiec et al., 2019). During development, both feedback and feed-forward signaling can regulate growth and maturation, processes that may be disrupted in CP in both MNs and muscle fibers. It is likely the loss of spinal interneurons, MNs and muscle fibers reduce coordination and strength in those with CP. Altered size and excitability of MNs is also a feature of other motor disorders including amyotrophic lateral sclerosis and spinal muscular atrophy (Quinlan et al., 2011, 2019; Gogliotti et al., 2012; Shoenfeld et al., 2014; Dukkipati et al., 2018). Since our data were collected from multiple MN pools throughout the spinal cord, from control and HI injured rabbit kits, relatively large variability in parameters was expected (Kanning et al., 2010), however consistent differences in electrical properties emerged in this data set. This suggests consistent changes are present across motor pools after injury and support further exploration of interventions for CP and other motor disorders that target spinal MNs. These treatments could include neuromodulators and therapies aimed at restoring balance between excitation and inhibition within spinal circuits for alleviation of spasticity.

### Neuromodulation

The exact causes of the changes in MN physiology observed here are unclear, but they could result from the increase in spinal monoamines that occurs after developmental HI injury in both rodents and rabbits (Bellot et al., 2014; Drobyshevsky et al., 2015). Serotonin is generally thought of as a neurotransmitter and neuromodulator, but developmental disruption in 5HT is associated with neurological disorders including autism, Rett syndrome, Down’s syndrome and, more recently, CP (Bar-Peled et al., 1991; Whittle et al., 2007; Bellot et al., 2014; Yang et al., 2014; De Filippis et al., 2015; Drobyshevsky et al., 2015; Muller et al., 2016; Wirth et al., 2017). Serotonin increases MN excitability in neonatal and juvenile mice, rats and guinea pigs (Wang and Dun, 1990; Ziskind-Conhaim et al., 1993; Hsiao et al., 1997, 1998), and likely has the same effect on rabbit MNs. Depolarization of the resting membrane potential, increased action potential firing through hyperpolarization of the voltage threshold and enhanced PIC, increased action potential height and reduction of high-voltage activated Ca^2+^ entry are all associated with 5HT receptor activation in neonatal and adult MNs (Elliot and Wallis, 1992; Larkman and Kelly, 1992; Lindsay and Feldman, 1993; Bayliss et al., 1995; Hsiao et al., 1997, 1998; Inoue et al., 1999; Gilmore and Fedirchuk, 2004; Li et al., 2006). Therefore increased 5HT could have a direct impact on excitability, though in HI rabbits the increase in 5HT was accompanied by decreased mRNA for 5HT_2_ receptors and increased mRNA for the SERT serotonin transporter (Drobyshevsky et al., 2015). In light of that finding, it is not clear that neurons remain responsive to 5HT. In the experiments here, all MNs were recorded in spinal cord slices incubated and perfused in standard oxygenated aCSF without any serotonergic drugs present. Therefore, HI MNs *in vivo* could show different levels of excitability since they would be in the presence of elevated 5HT, while our MNs were all recorded in the same standard physiological solution. Thus, the contribution of 5HT to the altered excitability observed here is restricted to its chronic effects on neuron development, namely morphological changes. Serotonin 5HT_1A_ and 5HT_2A_ receptor activation increases neurite outgrowth, dendritic branching, and spine formation (Bou-Flores et al., 2000; Fricker et al., 2005; Mogha et al., 2012), findings that align well with the present finding of increased dendritic length in the HI MNs. Future experiments will be needed to address the role of 5HT in enhancing MN excitability and its effects on synaptically-evoked action potentials. Synaptic events in dendrites would more strongly evoke PICs, though both altered dendritic morphology and elevated 5HT could dampen them.

### Possible Mechanism of Altered MN Output

The mechanism for increased MN activity and thus muscle stiffness may be due to delayed Na^+^ channel inactivation. In neonatal MNs, Na^+^ channels generate the majority of the PIC and account for action potential initiation/repetitive firing. Specifically, Nav 1.1, 1.2 and 1.6 type Na^+^ channels (Boiko et al., 2001, 2003; Rush et al., 2005) inactivate faster than Ca^2+^ channels that contribute to PICs (Perrier and Hounsgaard, 2003; Li et al., 2006). Short voltage ramps preferentially measure the Na^+^ PIC for this reason: Na^+^ channels inactivate quickly enough that even on the descending ramp of the short protocol, there is no longer a region of negative slope (see Figure 4). Changes in Na^+^ channel inactivation in adult MNs along with postnatal development of the longer-lasting Ca^2+^ PIC makes typical adult MNs display more negative ΔI values and longer-lasting PICs (Harvey et al., 2006; Li et al., 2006; Quinlan et al., 2011). In the neonatal rabbit MNs, sham controls showed positive ΔI values that are quite typical for neonates, while HI MNs showed significantly more negative values. This could be due to slower Na^+^ channel inactivation or increased contribution of Ca^2+^ channels to the PICs after injury. Interestingly the more depolarized resting potential found in HI severe MN would serve to increase Na^+^ channel inactivation. To fully determine the altered mechanism of aberrant firing after HI injury future studies into the biophysical properties of Na^+^ channels and maturation of Ca^2+^ channel expression must be pursued.

### Severity in Motor Deficits and Electrophysiology

Generally, unaffected and mildly affected MNs showed parameters that were intermediate between control MNs and severe MNs. There was only one category in which mildly affected MNs become statistically significantly different from sham controls (instantaneous firing frequency). Thus, electrophysiological changes were overwhelmingly in line with phenotype, suggesting aberrant MN properties contribute to the severity of the phenotype. It cannot be ruled out, however, that HI “unaffected” MNs may have a subtle phenotype that is not readily evident based on testing we performed here. Or perhaps abnormalities in these rabbits would develop in later in life: in humans CP patients, diagnosis of CP is not made until 18–24 months of life and the peak of spasticity occurs around 4 years of age (Hägglund and Wagner, 2008; Hadders-Algra, 2014; Novak et al., 2017). In the rabbit model, deficits have not been characterized past P18, and a detailed analysis of the progression of motor deficits from P0–18 is lacking. Future work is needed to assess the maturation of the MN properties in different groups, the potential contribution of delayed Na^+^ channel inactivation in CP, the progression of motor deficits with age, and the development of new therapeutic strategies that could target MNs.

### Postnatal Maturation and Injury

The present study only extended from postnatal day 0–5, but even within this narrow window, significant changes were apparent in MN electrical parameters. As MNs undergo postnatal maturation, they grow larger, with more complex dendritic arborizations, and gain the ability to fire action potentials at higher rates, as reviewed in Carrascal et al. (2005). We found that age had a significant effect on 5 s PIC amplitude (increasing with age), normalized PIC amplitude (both 5 and 16 s PIC/Cap increased with age), input resistance (decreasing with age), and action potential size, rate of rise and rate of fall (all increase with age). While neuron size increases during this period, the amplitude of the PIC typically increases more than proportionally (Quinlan et al., 2011). In other words, voltage-gated ion channels are being inserted into the membrane at a faster rate than the cell is increasing in size, resulting in an increased normalized PIC amplitude with age. We suspect this parallels the ability of MNs to fire action potentials at higher rates during postnatal development, and the acquisition of coordinated motor control and weight-bearing in developing animals.

## Conclusion

Changes in MN physiology after developmental injury are consistent with motor deficits in rabbits. This suggests not only brain injuries but also changes in the spinal cord contribute to impaired function in CP. Exploring both altered maturation of spinal neurons and loss of descending connectivity should be pursued to improve outcomes for individuals with CP.

This manuscript has been released as a Pre-Print at BioRxiv (Steele et al., 2019).

## Data Availability Statement

All datasets generated for this study are included in the article/Supplementary Material.

## Ethics Statement

The animal study was reviewed and approved by University of Rhode Island’s, Northwestern University’s and Northshore University Health System’s Animal Care and Use Committees.

## Author Contributions

PS, CC, MG, and KQ wrote the manuscript. PS, CC, LD, MW, and KQ performed the experiments and analyzed the data. AD, MG, and KQ planned the experiments and interpreted the results. PS, CC, and NK performed statistical analysis. All authors approved the content and assisted in revision and review of manuscript.

## Conflict of Interest

The authors declare that the research was conducted in the absence of any commercial or financial relationships that could be construed as a potential conflict of interest.
