# Supplementary material for: Altered Motoneuron Properties Contribute to Motor Deficits in a Rabbit Hypoxia-Ischemia Model of Cerebral Palsy
Source: Front Cell Neurosci. 2020 Mar 25;14:69. doi: 10.3389/fncel.2020.00069 (PMC7109297; doi:10.3389/fncel.2020.00069)
Supplement: Supplementary file 1 [file Data_Sheet_1.docx]

***Table S1 Action potential characteristics***

| ***Variable*** | ***Condition*** | ***Mean*** | ***SD*** | ***N*** | ***p*** |
| --- | --- | --- | --- | --- | --- |
| AP height past 0  (mV) | Sham control | 38.8 | 13.5 | 9 |  |
|  | HI unaffected | 39.2 | 7.6 | 12 | 0.499 |
|  | HI mild | 39.9 | 8.8 | 10 | 0.737 |
|  | HI severe | 40.5 | 8.6 | 8 | 0.413 |
| AP duration (ms) | Sham control | 1.6 | 0.4 | 9 |  |
|  | HI unaffected | 1.3 | 0.3 | 12 | 0.548 |
|  | HI mild | 1.3 | 0.4 | 10 | 0.877 |
|  | HI severe | 1.4 | 0.2 | 8 | 0.339 |
| AP rate of rise (V/s) | Sham control | 116 | 31 | 9 |  |
|  | HI unaffected | 117 | 20 | 12 | 0.947 |
|  | HI mild | 124 | 27 | 10 | 0.527 |
|  | HI severe | 115 | 19 | 8 | 0.940 |
| AP rate of fall (V/s) | Sham control | -46.4 | 14.7 | 9 |  |
|  | HI unaffected | -52.4 | 9.6 | 12 | 0.305 |
|  | HI mild | -57.7 | 18.1 | 10 | 0.071 |
|  | HI severe | -50.7 | 8.3 | 8 | 0.507 |
| AHP amp (mV) | Sham control | 12.4 | 3.2 | 7 |  |
|  | HI unaffected | 10.5 | 1.7 | 9 | 0.147 |
|  | HI mild | 12.7 | 3.3 | 7 | 0.843 |
|  | HI severe | 11.1 | 2.7 | 7 | 0.344 |
| AHP half amp dur (ms) | Sham control | 164 | 49 | 7 |  |
|  | HI unaffected | 122 | 55 | 9 | 0.217 |
|  | HI mild | 179 | 70 | 7 | 0.727 |
|  | HI severe | 165 | 93 | 7 | 1.000 |
| AHP tau (/s) | Sham control | .098 | .027 | 7 |  |
|  | HI unaffected | .098 | .077 | 9 | 0.985 |
|  | HI mild | .115 | .033 | 7 | 0.496 |
|  | HI severe | .079 | .027 | 7 | 0.496 |

*Significant difference to sham animals

***Table S2 Characteristics of Ih***

| ***Variable*** | ***Condition*** | ***Mean*** | ***SD*** | ***N*** | ***p*** |
| --- | --- | --- | --- | --- | --- |
| Sag (mV) | Sham control | 18.2 | 15.5 | 9 |  |
|  | HI unaffected | 13.9 | 9.4 | 12 | 0.339 |
|  | HI mild | 14.2 | 7.0 | 10 | 0.390 |
|  | HI severe | 14.1 | 5.5 | 8 | 0.408 |
| Rebound (mV) | sham control | 6.1 | 5.0 | 9 |  |
|  | HI unaffected | 6.1 | 3.2 | 12 | 0.996 |
|  | HI mild | 6.6 | 2.0 | 10 | 0.764 |
|  | HI severe | 6.2 | 2.4 | 8 | 0.954 |
| Sag % | Sham control | 48.4 | 32.6 | 9 |  |
|  | HI unaffected | 36.9 | 13.2 | 12 | 0.225 |
|  | HI mild | 39.6 | 20.5 | 10 | 0.373 |
|  | HI severe | 37.6 | 14.2 | 8 | 0.302 |
| Rebound % | Sham control | 16.9 | 9.8 | 9 |  |
|  | HI unaffected | 17.2 | 6.0 | 12 | 0.932 |
|  | HI mild | 19.2 | 10.2 | 10 | 0.546 |
|  | HI severe | 17.1 | 7.5 | 8 | 0.964 |

*Significant difference to sham animals
